# Implementation of a recovery-oriented model in a sub-acute Intermediate Stay Mental Health Unit (ISMHU)

**DOI:** 10.1186/s12913-016-1939-8

**Published:** 2017-01-03

**Authors:** Barry G. Frost, Megan Turrell, Ketrina A. Sly, Terry J. Lewin, Agatha M. Conrad, Suzanne Johnston, Srinivasan Tirupati, Kerry Petrovic, Sadanand Rajkumar

**Affiliations:** 1 School of Psychology, Faculty of Science and Technology, University of Newcastle, Callaghan, NSW 2308 Australia; 2 Centre for Brain and Mental Health Research, University of Newcastle, Callaghan, NSW 2308 Australia; 3 Hunter New England Mental Health, Newcastle, NSW 2300 Australia; 4 School of Medicine and Public Health, Faculty of Health and Medicine, University of Newcastle, Callaghan, NSW 2308 Australia; 5 Nurse Unit Manager (2010-2015), ISMHU, Hunter New England Mental Health, Newcastle, NSW 2300 Australia; 6 MH-READ Unit, Centre for Brain and Mental Health Research, Hunter New England Mental Health and the University of Newcastle, Callaghan, NSW 2308 Australia

**Keywords:** Health service evaluation, Inpatients, Mental disorders, Mental health services, Recovery, Rehabilitation, Serious mental illness, Sub-acute

## Abstract

**Background:**

An ongoing service evaluation project was initiated following the establishment of a new, purpose-built, 20-bed sub-acute Intermediate Stay Mental Health Unit (ISMHU). This paper: provides an overview of the targeted 6-week program, operating within an Integrated Recovery-oriented Model (IRM); characterises the clients admitted during the first 16 months; and documents their recovery needs and any changes.

**Methods:**

A brief description of the unit’s establishment and programs is initially provided. Client needs and priorities were identified collaboratively using the Mental Health Recovery Star (MHRS) and addressed through a range of in-situ, individual and group interventions. Extracted client and service data were analysed using descriptive statistics, paired t-tests examining change from admission to discharge, and selected correlations.

**Results:**

The initial 154 clients (165 admissions, average stay = 47.86 days) were predominately male (72.1%), transferred from acute care (75.3%), with schizophrenia or related disorders (74.0%). Readmission rates within 6-months were 16.2% for acute and 3.2% for sub-acute care. Three MHRS subscales were derived, together with stage-of-change categories. Marked improvements in MHRS Symptom management and functioning were identified (*z-change = −*1.15), followed by Social-connection (*z-change = −*0.82) and Self-belief (*z-change = −*0.76). This was accompanied by a mean reduction of 2.59 in the number of *pre-action* MHRS items from admission to discharge (*z-change* = 0.98). Clinician-rated Health of the Nation Outcome Scales (HoNOS) improvements were smaller (*z-change* = 0.41), indicative of illness chronicity. Staff valued the elements of client choice, the holistic and team approach, program quality, review processes and opportunities for client change. Addressing high-levels of need in the 6-week timeframe was raised as a concern.

**Conclusions:**

This paper demonstrates that a recovery-oriented model can be successfully implemented at the intermediate level of care. It is hoped that ongoing evaluations support the enthusiasm, commitment and feedback evident from staff, clients and carers.

**Electronic supplementary material:**

The online version of this article (doi:10.1186/s12913-016-1939-8) contains supplementary material, which is available to authorized users.

## Background

### Recovery frameworks

In the absence of research data, the consumer movement identified a range of issues profoundly influencing their mental health (MH) and role functioning, including hopelessness, dependence, lack of control and loss of sense of self, which has led to the emergence of a positively focused recovery-oriented model [1]. This model assumes that people with a serious mental illness (SMI) have the capacity to develop a stronger sense-of-self and mastery and to pursue a life not defined by their illness. Indeed, research has shown 50-70% of people with a SMI experience high levels of recovery and improvement [2], with the frequency and periods of wellness increasing with age [1].

While the term ‘recovery’ is used in varied ways [1, 3–6], it nevertheless conveys an unequivocal message of a better future. With respect to MH, recovery may occur on a personal and/or a clinical level; however, until recently the focus has been almost exclusively on clinical recovery [7]. Typically, *clinical recovery* is “… *primarily defined by mental health professionals and pertains to a reduction or cessation of symptoms and restoring social functioning*” ([8], p. 2), while *personal recovery* refers to “… *being able to create and live a meaningful and contributing life in a community of choice with or without the presence of mental health issues*” ([9], p. 4).

Personal recovery has been conceptualised as an active process, focused on the achievement of a satisfying and fulfilling role, irrespective of symptoms [10]. That is, recovery reflects a shift from a passive to an active sense-of-self, from dependent to self-determining and from isolation to connectedness [6]. Although the recovery pathway is often complex and non-linear, hope is critical in shaping and sustaining improvements and social inclusion [11, 12].

Importantly, recovery is both a process and an outcome, and consumer descriptions (eg, of personal wellbeing and social inclusion) need to be incorporated within recovery-oriented services to improve service outcomes [13]. Encouraging clients to assume greater independence is axiomatic to ‘Clinical Rehabilitation’ [14] and consistent with the key elements of personal recovery. Although processes and outcomes can be difficult to distinguish, the greater the symptomatic and functional improvement, the higher the expected levels of hope, empowerment, self-responsibility and autonomy [1].

### Recovery-oriented service provision

Australian MH services are currently in transition, including national reviews of programs and services [15], development of a new MH Care Classification [16], and introduction of health service wide Activity Based Funding [17]. Concurrently, attempts have been made to formulate a ‘*national framework for recovery-oriented mental health services*’ – to provide guidance for practitioners and service providers [18] and to outline relevant research, conceptual and policy underpinnings [9]. Similar ‘recovery-focused’ frameworks have been developed elsewhere [5, 19]. For example, in the United Kingdom templates and guides have been produced for many aspects of MH service provision, including ‘rehabilitation services’ (eg, [20, 21]), for which the contemporary definition is: “… *recovery-oriented service for people with disabilities associated with longer-term mental health problems*” ([21], p. 10).

Recovery-oriented service provision can occur in a multitude of forms. “*The notion of recovery does not equate with a particular model of care … or service setting*”; rather, ‘recovery’ can be viewed as “*an overarching philosophy that encompasses … self-determination, self-management, personal growth, empowerment, choice and meaningful social engagement*” ([8], p. 2). We have adopted the following definition: *recovery-oriented service delivery* “*… is centered on and adapts to people’s aspirations and needs, rather than people having to adapt to the requirements and priorities of services*” and it has a “…*responsibility to provide evidence-informed treatment, therapy, rehabilitation and psychosocial support that assist in achieving the best outcomes for people’s mental health, physical health and wellbeing*” ([9], p. 26).

Five ‘practice domains’ (covering 17 capabilities) have been identified within the proposed national recovery-oriented framework for MH services: promoting hope and optimism; adopting a person-centred and holistic perspective; supporting personal recovery; developing an appropriate organisational culture and skilled workforce; and action on social inclusion and social determinants [18]. These domains and capabilities are largely consistent with the synthesis of recovery-oriented practice guidance provided by Le Boutillier et al. [19].

In developing recovery-oriented but often time limited MH programs, some of the key elements requiring consideration are: methods for identifying current client needs and priorities; selection of appropriate assessment and outcome measures; devising tailored evidence-based interventions (EBIs); engagement with families and carers; and strategies for fostering ongoing community/social linkages. The task of improving MH can also be expressed in other ways. For example, many researchers propose that outcomes can be improved through better access to psychosocial EBIs [22–27]. Evidenced-based practices are consistent with the recovery model provided that the client is encouraged and supported to assume responsibility in the domains of need, at the earliest possible opportunity. Providing adequate staff training in the flexible delivery of recovery-oriented programs is also an ongoing issue for mental health services [28, 29].

### The current study

Elsewhere we have described a general recovery-oriented framework for MH services, which has been characterised as an Integrated Recovery-oriented Model (IRM) [14]. ‘Clinical Rehabilitation’ approaches, practices and practitioners are viewed as pivotal, utilising EBIs and processes to help achieve and maintain optimal functioning and to promote recovery, self-agency and social inclusion. The IRM was designed to address the recovery needs of people with a SMI by improving access to a suite of EBIs provided in an integrated, multi-layered service context that: 1) *remediates symptoms and reinstates hope* (primarily via acute/emergency MH services; 2) *restores and rebuilds competencies* (primarily via specialised MH Clinical Rehabilitation services); and 3) provides *opportunities to reconnect* with place and society (primarily via General Practitioners, and Community Managed and Non-government Organisations providing integrated community services) [14].

Almost by definition, it is not possible to formulate a ‘one-size fits all’ strategy for providing recovery-oriented MH services, with clients at different recovery stages needing access to different EBIs (ie, the ‘right care’ at the ‘right time’) [30]. In this paper, we illustrate the application of recovery-oriented practices at the ‘intermediate’ level of care; that is, within a specialised sub-acute inpatient MH context (see Additional file 1 for detailed definitions), in which each client’s strengths, capabilities and needs can be observed and adequately assessed, and they can be progressively exposed to more intensive, personalised and group interventions – with the ultimate goal of stabilising and improving their recovery trajectory.

The aims of this paper are three-fold: 1) to briefly document the establishment of the Intermediate Stay Mental Health Unit (ISMHU) and the implementation of an IRM within a targeted 6-week sub-acute inpatient program; 2) to characterise the clients admitted during the unit’s first 16 months of operation; and 3) to quantify their recovery needs and priorities on admission and any changes during the admission.

## Methods

### Study design

The current paper is part of an ongoing service evaluation project, with data drawn primarily from regional clinical records associated with the participating unit, and all processing and analysis undertaken by MH service staff. This project was viewed as an internal, low risk study and exempted from the requirement for a formal regional ethics committee application.

### Intermediate Stay Mental Health Unit (ISMHU)

#### Establishment, service context, staffing and training strategy

In 2005, New South Wales Health commenced planning for the establishment of a number of 20-bed sub-acute/non-acute inpatient units [30, 31]. Improved access to recovery-focused rehabilitation services that are highly integrated and rigorously evaluated was the primary goal. To-date, eight units have been established (totalling 140 beds), although they vary in their models of care and their typical lengths of stay (eg, [32]).

The new, purpose-built, 20-bed, sub-acute ISMHU within Hunter New England Mental Health services (Newcastle, Australia) opened in November 2010, with the specific intention of providing a planned, 6-week, recovery-focused intervention program. To optimise access, the criteria for ISMHU admission and transfer were kept to a minimum. The primary focus was the recovery needs and priorities of adults aged 16–65 years with a SMI who were *not* acute and considered to be at low risk.

The ISMHU service model and 6-week program were developed under the stewardship of a representative committee. In broad terms, this program was designed to operate within the framework of an IRM for MH services that seeks to support and promote ‘remediation, restoration and reconnection’ [14]. As noted above, specialised Clinical Rehabilitation approaches were regarded as central (eg, utilising recovery-focused EBIs and processes). The specific goals of the ISMHU program were: to improve psychological and physical wellness; enhance personal and interpersonal coping skills; improve daily functioning; enhance social, family and community supports; and, thereby, to encourage a new or higher sense of self-management and social inclusion through the attainment of socially valued roles.

With regards to staffing, the ISMHU program also departed somewhat from traditional Australian approaches to MH service delivery, including adjustments to recruitment strategies, staffing profiles, training programs and roster arrangements. Some of these specific elements included: staff recruitment against a predetermined set of values and skills (eg, openness, empathy, support for responsible risk taking); a broader mix of allied health staff; a senior rehabilitation clinician functionally ‘embedded’ in acute inpatient and community-based MH services, to ensure appropriate and timely ISMHU referrals; a Non-government Organisation peer-link worker liaising closely with community-based day programs; and a range of work day and after-hours programs. See Additional file 1 for further information about ISMHU’s establishment, service context (recovery-oriented service delivery and sub-acute classifications), staffing and training strategy.

#### Program

To underpin the ISMHU recovery-oriented approach, the Mental Health Recovery Star (MHRS), a collaborative planning, review and assessment tool was adopted [33]. Essentially, the MHRS provides a straightforward framework for conducting a structured conversation with clients, which, in the current context, was undertaken during the first and last few days of the ISMHU stay. To support the collaborative development of an intervention plan built around the MHRS domains, a MHRS program guide was developed. The programs were collaborative, goal-focused, evidence-based and motivational, promoted generalisation, accommodated different learning styles and were structured to support achievement. The guide mapped the goals and interventions available in each of the ten potential domains and included core programs and electives. Importantly, time limited programs can only address some of the relevant recovery domains for each person; consequently, in addition to identifying medium- to longer-term recovery-oriented goals, collaborative assessments using the MHRS were used to help set priorities for each person for the subsequent 6 weeks.

Key elements of the ISMHU program are summarised in Table 1. These elements included opportunities for in situ interventions as well as more formal individual and group programs, which could be unit and/or community-based; where possible, they were also coupled with interventions provided through community-based rehabilitation teams. For clients who were unable or unwilling to participate in core programs, activity-based engagement programs were also offered. Family interventions were offered on an ongoing basis in both group and individual formats.

**Table 1:** Program: collaborative pathways to recovery, resilience and independence, utilising MHRS framework

Client priorities	Assessment ^a^	In situ and individual interventions	Client and family/carer groups	Community support linkages
• Mental health & self-reliance • Physical health & self-care • Daily living skills & self-management • Social networking & community participation • Access to education & employment • Healthy meaningful relationships • Coping strategies to reduce risk of addictive behaviours • Skills to self-manage daily responsibilities • More satisfying sense of self • Improved sense of hope & trust	• Insight, coping skills, situational factors/capacity • Stage-of-change • Physical health/status • Personal self-care skills (eg, diet, lifestyle, dental, podiatry & medical) • Domestic self-care & community skills • Social skills • Vocational & educational support needs • Sexual health • Cognitive functioning • Psychological health • Comorbidity issues • Addictive behaviours • Adherence skills • Quality of relationships • Day-to-day functioning	• Positive recovery focus reinforces strengths • Encourages coping strategies, self-advocacy & positive risk taking • Service provision promotes self-reliance, self-care & positive role modelling • Milieu encourages self-respect & maintenance • Supportive culture reflects individual & social values • Facilitates trusting relationships with staff • Motivational interviewing • Cognitive behaviour therapy & counselling • Skills development & cognitive remediation • Behavioural interventions • Relapse prevention • Self-esteem building • Social activities • Education: mental health, medication & lifestyle	• Managing mental health ^b^ • Medication • Goal setting/review ^b^ • Shopping/meal planning^b^ • Healthy lifestyles • Budgeting • Relaxation/stress management ^b^ • Social skills, community access BBQ, hobby starters^b^ • Wellness plan • Drug & alcohol education • QUIT interventions • Vocational education training & employment • Recovery Star discussion • Discharge planning • Family connection ^c^ • Mental health education ^c^	• Accommodation services (eg, supported housing) • Education & employment services • Community, family & welfare services (eg, Community access, Centrelink, relationship & financial counselling, meals on wheels, ARAFMI, clubs, volunteer programs) • Mental health & substance use services (eg, Supported Recovery, Community Managed and Non-government Organisations, community mental health, early psychosis, psychiatrists, allied health) • Health services (eg, GP, dietary, dental, sexual health, recreational & cultural) • Internet services (eg, SANE Australia, Black Dog Institute)

*Note*: *MHRS* Mental Health Recovery Star, *ARAFMI* Association of Relatives And Friends of the Mentally Ill

^a^Conducted to assist in building a comprehensive recovery plan

^b^Group programs offered on repeat occasions per week

^c^Group programs offered outside of usual business hours to facilitate optimal carer/family involvement

### Measures

#### Mental Health Recovery Star (MHRS)

The MHRS [33], developed using a bottom-up approach [34], was designed to be a collaborative tool for assessing and discussing recovery experiences, needs and priorities across ten potential domains, and to guide recovery plans and interventions. Each domain is underpinned by a detailed stage-of-change hierarchy [35] and graphically represented as a ladder (from 1 to 10) within a ten-pointed star; ladder steps are characterised separately for each domain but also labelled generically as: Stuck (1–2); Accepting help (3–4); Believing (5–6); Learning (7–8); and Self-reliance (9–10) [33].

The MHRS was collaboratively completed by individual clients with guidance from the clinician coordinating their care. All available ISMHU-related data for the MHRS was downloaded from the associated online program and supplemented by a manual search of clinical records (for additional completed MHRS forms). Where possible, two sets of ratings were identified, one within a few days of admission and one near ISMHU discharge; on average (*N* = 94), the interval between these ratings was 37.9 days (SD = 17.4).

The MHRS has demonstrated high internal consistency in UK samples (Cronbach’s α = 0.85) [36] and good test-retest reliability at twelve days (ICC’s >0.7), as well as high acceptability and ease of use [37]. As detailed in our preliminary MHRS dimensionality analyses (see Additional file 2, *N* = 228 sets of ratings), a single factor solution does a reasonably good job at accounting for variation among the item ratings (Cronbach’s α = 0.88), providing strong justification for forming an overall MHRS score based on (averaging) the 10 items. In addition, it also seems reasonable to construct three correlated MHRS subscale scores, which may be particularly useful for examining change and differential predictive utility: ‘Symptom management and functioning’ (averaging four domains: Physical health and self-care; Managing mental health; Work; and Living skills) (Cronbach’s α = 0.78), ‘Self-belief’ (averaging four domains: Addictive behaviour; Identity and self-esteem; Trust and hope; and Responsibilities) (Cronbach’s α = 0.74); and ‘Social connection’ (averaging two domains: Relationships; and Social networks) (Cronbach’s α = 0.69).

#### Routine service outcome measures

Clinical and basic demographic data for all ISMHU admissions were obtained from electronic clinical records via the regional Inpatient Management System (IPMS). Selected assessment measures, routinely collected as part of the national outcomes set [38], were obtained via the regional Clinical Information and Management Exchange (CHIME), including: the Health of the Nation Outcomes Scales (HoNOS; [39]); and the Kessler psychological distress scale (K10; [40]). Twelve items are assessed in the HoNOS: problems with behaviour (3 items); impairment (2 items); symptoms (3 items); and social functioning (4 items). Items are rated on a 5-point scale ranging from (0) ‘no problem’ to (4) ‘severe to very severe problem’, with higher scores indicating poorer mental health [39]. Admission and discharge HoNOS ratings were often completed by different ISMHU clinicians. The HoNOS has displayed good construct and predictive validity, with adequate test-retest and inter-rater reliability and sensitivity to change [41, 42]. The K10 was completed by individual clients at ISMHU, as a measure of general psychological distress (during the last month); it is a 10 item measure, with higher scores indicating greater distress (range 10–50), which has been shown to have consistent psychometric properties across a range of samples [40].

### Data analysis

For the current analyses, the relevant data collection window was from the opening of ISMHU in November 2010 up until March 31st, 2012. IBM SPSS statistical software (Version 22.0; Armonk, NY, USA) was used to analyse the aggregated datasets. Many of the statistics reported here are descriptive, with paired t-tests used to examine change scores from admission to discharge, and bivariate correlations used to examine relationships between these change scores. As a partial control for the number of statistical tests, the threshold for statistical significance was set at *p* < 0.01.

## Results

### Client characteristics

ISMHU commenced operation at 50% capacity, with the remaining beds opened prior to March 2011. By the end of March 2012, the unit had received 165 admissions from 154 clients. Table 2 details the socio-demographic and clinical characteristics of these clients, who averaged 37.25 years of age, were predominantly male (72.1%) and transferred from acute inpatient units (75.3%).

**Table 2:** Client characteristics (November 2010 to March 2012, *N* = 154 clients)^a^

Socio-demographic and clinical characteristics	*N*	%
Age (Mean = 37.25 years, SD = 10.20):		
Under 25	17	11.0
25-39	73	47.4
40-54	57	37.0
55+	7	4.5
Gender		
Male	111	72.1
Female	43	27.9
Referral source		
Acute inpatient facility	116	75.3
Community	38	24.7
Legal status		
Voluntary	87	56.5
Involuntary^c^	67	43.5
Symptom severity on admission		
HoNOS (*N* = 114, Mean = 9.19, SD = 6.46):		
Mild (0–9)	65	57.0
Moderate (10–12)	19	16.7
Severe (>13)	30	26.3
Current distress on admission		
K10 (*N* = 79, Mean = 22.63, SD = 10.01)		
Low (10–15)	22	27.8
Moderate (16–21)	23	29.1
High (22–29)	13	16.5
Very high (30–50)	21	26.6
Any ICD-10 discharge diagnosis of:^b^		
Schizophrenia or related	114	74.0
Anxiety or related	68	44.2
Substance misuse	64	41.6
Depression	38	24.7
Bipolar disorder	22	14.3
Personality disorder	12	7.8
Other mental health problems	15	9.7
Psychosocial problems	146	94.8
Physical health problems	135	87.7
Length of stay (Mean = 47.86 days, SD = 24.16)		
Early exit (0 to 7 days)	8	5.2
Shorter than planned (8 to 40 days)	33	21.4
As planned (41–60 days)	77	50.0
Longer than planned (Over 60 days)	36	23.4
Discharge destination		
Community	138	89.6
Acute Inpatient facility^c^	16	10.4
Readmission within 6 months to		
Acute inpatient unit	25	16.2
Sub-acute inpatient unit	5	3.2

*Note: HoNOS* Health of the Nation Outcome Scales (12 items), *K10*, Kessler psychological distress scale

^a^154 clients with 165 admissions (9 clients had multiple admissions)

^b^At any stage during this admission sequence, including previous unit (if transferred)

^c^15 of these 16 clients were initially referred to ISMHU from an acute inpatient facility

Consistent with expectations, 56.5% of admissions were voluntary, with 74.0% having an ICD-10 diagnosis of schizophrenia or a related disorder. The next most common diagnoses were anxiety or a related disorder (44.2%), substance misuse (41.6%) and depression (24.7%); the vast majority of clients also reported psychosocial (94.8%) and/or physical health (87.7%) problems. On admission, the majority of clients (57.0%) had current functioning ratings in the mild category (0–9) on the HoNOS, with a similar percentage (56.9%) experiencing low or moderate distress (10–21) on the K10.

The average length of stay was 47.86 days, with 50.0% staying for 41 to 60 days. For those staying longer than 60 days (23.4%), the major reason was the complexity and/or acuity of issues identified. For 10.4% of clients the discharge destination was an acute inpatient facility. During the first 16 months, bed occupancy was 80.6% (ignoring client leave periods) and the average number of leave days was 6.75 (median of 5.00); 83.8% of admissions were associated with at least one leave period, with the first period commencing at a median of 25 days from admission. Although planned or transitional leave arrangements carry a service cost, the benefits accrued in supporting social connectedness and promoting confidence and self-sufficiency (as clients transitioned from a structured environment to community living) were considered to more than offset these costs. In the 6-month period following the index admission, 28 clients had a subsequent readmission, with 16.2% (25/154) admitted to an acute unit and 3.2% (5/154) re-admitted to ISMHU (2 individuals had both an acute and sub-acute component to their subsequent stay).

### Initial feedback

As part of the initial service review, ISMHU staff from across all disciplines volunteered to provide feedback on the model of care and unit performance; staff feedback sessions were held between February and March 2012, with 14 staff participating (or approx. 54%). They identified a number of achievements, as well as areas for improvement. In particular, staff valued: the model of care; client choice and self-determination; the multidisciplinary, holistic approach; quality of groups and education sessions; client review processes; opportunities to support change; significance of the work undertaken; interagency connections, family and carer involvement; discharge practices; and ISMHU’s built environment. Medical staff were appreciative of the opportunity to work intensively with clients in a supportive timeframe.

The feedback from clients, carers and families has been both formal and informal. A review of feedback collection methods has suggested that more reliable and effective methods need to be developed. Two examples of written feedback were: *“What can we say when thank you just isn’t enough! I can’t tell you how grateful I am to each and every one of you who cared for [person’s name] while he was a patient at ISMHU”;* and *“Thank you for being flexible, not serious and not being negative. Thanks for all the happiness and plenty of smiles. I had forgotten what it felt like to be normal and thank you for your support.”*

There were also several suggested areas for improvement: reconciling the recovery-oriented approach with involuntary admissions; management of clients with elevated levels of risk; recovery-oriented training and supervision; insufficient day shift nursing staff; care coordination; and insufficient time during 6-weeks to effectively intervene. Some staff also suggested there should be greater flexibility in length of stay (eg, 9-weeks or a split unit with 6- and 9-week stays).

### MHRS profiles

To date, the MHRS does not have an agreed reporting format; however, we encourage other researchers and clinicians to explore usage of the subscales detailed in Additional file 2, as well as the stage-of-change categories in Table 3. For convenience, we chose a simple additional metric for characterising MHRS, namely the percentages below 6 (‘Pre-action’), from 6 to below 9 (‘Action’), and between 9 and 10 (‘Self-reliant’ stage). To support our descriptive label for the first cut-point, it should be noted that the generic MHRS descriptor for step 5 includes: “… *The next step is to act on this change*” [33]. Arguably, scores categorised as ‘pre-action’ require some form of intervention or other input from services or support networks to help stimulate change and promote recovery.

**Table 3:** Mental Health Recovery Star (MHRS) profiles on admission (*N* = 131)

MHRS subscale/items	Mean (SD)	Median	Stage-of-change (%)^a^		
			Pre-action	Action	Self-reliant
Symptom management & functioning:	5.69 (1.48)	5.75	55.7	42.7	1.5
(2) Physical health and self-care	6.11 (2.05)	6.00	42.7	45.0	12.2
(1) Managing mental health	5.43 (1.86)	5.00	58.8	35.9	5.3
(5) Work	4.66 (1.86)	5.00	71.0	26.7	2.3
(3) Living skills	6.56 (2.32)	7.00	37.4	35.1	27.5
Self-belief:	6.49 (1.49)	6.50	34.4	59.5	6.1
(7) Addictive behaviour^b^	6.72 (2.49)	7.00	33.6	39.7	26.7
(9) Identity and self-esteem	5.72 (2.13)	5.00	50.4	37.4	12.2
(10) Trust and hope	5.95 (2.08)	6.00	44.3	45.0	10.7
(8) Responsibilities^b^	7.58 (1.96)	8.00	18.3	45.0	36.6
Social connection:	5.18 (1.93)	5.00	63.4	32.8	3.8
(6) Relationships	5.34 (2.34)	5.00	57.3	32.1	10.7
(4) Social networks	5.02 (2.34)	5.00	61.8	26.7	11.5
Overall score (10 items)	5.98 (1.31)	6.00	49.6	48.9	1.5

*Note*: Item numbers from the MHRS are shown in brackets; see Additional file 2 for subscale development

^a^Item or subscale score classification: Pre-action <6; Action ≥6 & <9; Self-reliant 9–10; Number of *pre-actio*n items: Mean = 4.76 (SD = 2.60)

^b^A score of 10 is given for Addictive behaviour and/or Responsibilities if no difficulties are reported

Table 3 summarises MHRS profiles for the 131 clients with available admission data. The items revealing the highest need/priority (ie, based on lower mean ratings) were: Work (or work-like activity), Social networks, Relationships, Managing MH and Identity and self-esteem; all of these items had median MHRS ratings of 5.00. Clients typically required support in, at least, four or five domains (mean number of *pre-action* items = 4.76).

### Improvements during admission

Admission to discharge change profiles for the HoNOS, MHRS subscales and overall score are displayed in the left-hand columns of Table 4; these analyses were based on all available data pairs. While all analyses demonstrated significant improvements across the course of the 6-week admission, there were more marked improvements on the MHRS Symptom management and functioning subscale (mean *z-change* = −1.15) compared with, for example, the MHRS Self-belief subscale (mean *z-change* = −0.76). Clinician rated HoNOS scores, based on 82 admission and discharge pairs, displayed the smallest change (mean *z-change* = 0.41), perhaps reflective of the sub-acute but nevertheless chronic and debilitating nature of the clients’ illnesses. Using the simplified HoNOS ‘meaningful outcome’ classification method described by Parabiaghhi et al. [43]: 42.7% (35/82) ‘improved to mild’ (ie, a HoNOS reduction ≥ 4, and shifting into the mild category); 52.4% were ‘stable’ (33 with ‘mild’, 5 with ‘moderate’ and 5 with ‘severe’ HoNOS scores at discharge); and 4.9% (4/82) ‘deteriorated’ (ie, HoNOS worsening by ≥ 4). There was a relative absence of correlations between change scores from clinicians and clients (although different measures were used; see the right-hand columns of Table 4), suggesting that they tended to have different perspectives. There was also a substantial improvement in stage-of-change, as evidenced by a reduction in the number of *pre-action* MHRS items from admission to discharge (mean change = 2.59 items; mean *z-change* = 0.98).

**Table 4:** Change during the admission for selected measures and correlations between change scores

Measure (Number of items)	Admission	Discharge	Change		Selected correlations between change scores (see Row labels for measures)			
	Mean (SD)	Mean (SD)	Mean (SD) *[z-change]*	Paired *t*-test	II	III	IV	V
Symptom Severity – HoNOS (*N* = 82):								
I. Overall score (12)	8.66 (5.90)	5.96 (5.40)	2.70 (6.53) *[0.41]*	3.74**	-.250	-.289^#^	-.112	-.254
MHRS (*N* = 94):								
II. Symptom management & functioning (4)	5.74 (1.46)	7.15 (1.40)	−1.41 (1.23) *[−1.15]*	−11.16**		.679**	.676**	(.899)
III. Self-belief (4)	6.61 (1.51)	7.61 (1.54)	−1.01 (1.32) *[−0.76]*	−7.41**			.626**	(.892)
IV. Social connection (2)	5.26 (1.89)	6.78 (1.97)	−1.53 (1.85) *[−0.82]*	−7.90**				(.844)
V. Overall score (10)	5.99 (1.28)	7.26 (1.37)	−1.27 (1.22) *[−1.04]*	−10.09**				
Number of *pre-action* MHRS items^a^	4.53 (2.57)	1.95 (2.48)	2.59 (2.63) *[0.98]*	9.52**				

*Note*: *HoNOS* Health of the Nation Outcome Scales (12 items: Clinician rated), *MHRS* Mental Health Recovery Star (Collaboratively completed: Clinician/Client)

^a^Count of MHRS item scores <6; ^#^(trend, *p* < 0.05); *p < 0.01; **p < 0.001

## Discussion

### Implementation of targeted 6-week recovery-focused program

We have contextualised the ISMHU program within an overarching model of care for MH services (ie, IRM, incorporating specialised Clinical Rehabilitation approaches) [see 14]. This model was developed in parallel with the emerging national recovery-oriented frameworks in Australia [9, 18] rather than being a direct product of those initiatives.

Establishing a new unit provided a rare opportunity to develop and implement an innovative recovery-oriented model at the intermediate (sub-acute) level of care. It seems almost self-evident that if the recovery needs and priorities of people with high levels of unmet need are addressed (within a targeted, evidence-based program) and within a positive environment, focused on building independence and social inclusion, then considerable MH and psychosocial benefits are likely to accrue. Notwithstanding, developing and evaluating MH services that support and promote recovery is challenging.

The initial success of the ISMHU program has been reflected in the enthusiasm, commitment and feedback received from staff, clients and carers. The concerns expressed by staff during the initial service review may relate to the complexities in addressing the high levels of need within a limited timeframe but with a staffing budget more aligned with traditional non-acute services. It is also possible that the staff concerns may have more to do with the broader acceptance and establishment of the unit than with the underlying model of care. Many staff had very high expectations, even though few, if any, had experience at this level of care.

The benefits of promoting client led recovery in an environment that affords a unique opportunity to address high levels of need, and in a service context that is more consonant with staff, client and family expectations, may be considerable. Stepping outside the orthodoxy may raise many issues, but if the initial feedback can be supported by further research and evaluation evidence then it has certainly been a step worth taking. Individualised approaches to recovery have been applied to vocational rehabilitation, and found to have positive effects not only for employment but on clinical outcomes [44].

The program delivered at ISMHU should probably be viewed as one component, albeit a useful one, within the armoury of potential recovery-oriented intervention programs for individuals with a SMI. It was targeted at the intermediate level of care, program driven but personalised and framed within a broader IRM for MH services. As a planned, limited duration inpatient program, it also facilitated a more comprehensive assessment of current competencies and strengths, enabled trial leave periods and provided opportunities to forge or strengthen community linkages. However, it should also be acknowledged that this particular, hospital-based, sub-acute recovery-oriented program is not likely to be suitable for everyone, nor, for that matter, are collaborative assessment tools such as the MHRS [45].

### Recovery needs, priorities and changes

The MHRS plays a central role within ISMHU, providing a basis for: calibrating stage-of-change; prioritising intervention plans; characterising the nature of the ISMHU programs on offer (with respect to MHRS domains); and for analysing change. Moreover, the MHRS was generally well received by clients and staff – mirroring experiences elsewhere [45, 46]. The newly identified MHRS subscales (see Additional file 2) may also prove useful in both clinical and research settings.

The MHRS admission profiles in Table 3 are highly consistent with those reported in a UK study of 203 clients with moderate to severe MH problems attending a range of community-based MH services [36]. These domain profiles are also similar to the needs identified in a population of severely mentally ill in Sweden over a 10-year period [47]. Mean improvement in overall MHRS scores (of 1.27) was similar to that reported for interventions delivered over a three-month period by specialised recovery clinicians [46]. Improvements on the MHRS Symptom management and functioning subscale were more marked, but all of the subscales displayed significant improvement from admission to discharge (see Table 4).

Improvements on the MHRS Self-belief subscale were somewhat smaller, possibly suggesting that these domains are harder to shift; however, mean scores on this subscale were also higher on admission, with potentially less need or room for change. Importantly, at discharge, the mean number of MHRS items remaining at the *pre-action* stage was only 1.95 (out of 10), suggestive of a substantial change in recovery trajectories or potentially a ‘turning point’ associated with the ISMHU program.

The MHRS subscale change scores for Symptom management and functioning, Self-belief and Social connection were all significantly moderately correlated (0.626–0.679, see Table 4); however, they relate to one ‘change timeframe’ (ie, across the course of a targeted 6-week admission). It is anticipated that differential change patterns will be more likely to occur over a longer period of time, as different external factors influence different recovery domains. Consequently, evaluations of underlying recovery components should be considered when examining sustained change, using MHRS subscale and overall scores, together with other psychosocial functioning indices and evaluation methods.

With respect to current functioning, the ISMHU clinicians rated the majority of clients within the ‘mild’ HoNOS category on admission, which is consistent with the unit’s sub-acute focus; notwithstanding, there were detectable HoNOS changes at discharge, with two-fifths (42.7%) displaying clinically meaningful improvement [43]. Conversely, one-in-ten (10.4%) ISMHU clients were transferred back to an acute inpatient facility, which is comparable to the 6.0% to 10.7% rates from other Australian units [48, 49].

### Study limitations

Limitations include: fluctuations in available service data (eg, 154 admissions, 131 MHRS admission ratings, 94 admission/discharge data pairs); no post-discharge assessments; lack of a comparison group; and reliance on data from a single service. The absence of correlations between change scores from clinicians and clients (using different measures) suggests that they tend to have different perspectives; indeed, clients’ and clinicians’ perceptions of recovery have previously been found to differ [50]. Further strengthening of the overall measurement approach may be required. For example, a wider variety of domains may need to be assessed from multiple perspectives (eg, current symptoms from a client perspective, and perceived hope and preparedness to change from a clinician-based perspective).

## Conclusions

This paper provides an initial characterisation of a new sub-acute inpatient MH unit and 6-week program. In broad terms, the findings are reassuring (eg, successful ISMHU establishment; implementation of targeted, acceptable and valued programs; detectable MHRS and HoNOS improvements); however, these findings are also preliminary and largely descriptive. Potential future directions include: examination of relationships between the intensity of program engagement during the ISMHU stay and personal and service outcomes; inclusion of post-ISMHU follow-up assessments; evaluation of other factors affecting and reflecting recovery processes (eg, engagement, self-determination); and further analysis of the impact of ISMHU admissions on recovery trajectories (eg, symptom profiles, service contacts and admissions during the two years either side of the index ISMHU admission). In the meantime, implementation of our broader IRM within a sub-acute setting has undoubtedly strengthened our services’ recovery-oriented practices for individuals with a SMI.

## Additional files

Additional file 1: The Intermediate Stay Mental Health Unit - Establishment, Service Context, Staffing and Training Strategy. (DOCX 45 kb)
Additional file 2: Mental Health Recovery Star (MHRS) Dimensionality – Explorations using data from the Intermediate Stay Mental Health Unit. (DOCX 84 kb)

## Abbreviations

CHIME: Clinical information and management exchange
EBIs: Evidence-based interventions
HoNOS: Health of the Nation Outcomes Scales
ICD-10: International Classification of Diseases
IPMS: Inpatient management system
IRM: Integrated recovery-oriented model
ISMHU: Intermediate stay mental health unit
K10: Kessler psychological distress scale
MH: Mental health
MHRS: Mental health recovery star
SMI: Serious mental illness
