# Supplementary material for: Implementation of a recovery-oriented model in a sub-acute Intermediate Stay Mental Health Unit (ISMHU)
Source: BMC Health Serv Res. 2017 Jan 3;17:2. doi: 10.1186/s12913-016-1939-8 (PMC5210223; doi:10.1186/s12913-016-1939-8)
Supplement: Additional file 1: — The Intermediate Stay Mental Health Unit - Establishment, Service Context, Staffing and Training Strategy. (DOCX 45 kb) [file 12913_2016_1939_MOESM1_ESM.docx]

**Supplementary Document 1 (December, 2016): *“The Intermediate Stay Mental Health Unit - Establishment, Service Context, Staffing and Training Strategy (ISMHU, Hunter New England Mental Health Services, Newcastle, Australia)”***

A new, purpose-built, 20-bed, sub-acute Intermediate Stay Mental Health Unit (ISMHU) opened within Hunter New England Mental Health services (Newcastle, Australia) in November 2010. This supplementary document briefly describes the establishment of this unit, and associated staffing and training strategies (see the main text for program components and details). Some brief background information about the Australian mental health service context is provided first.

***Australian Mental Health Service Context***

As noted in the main text, Australian mental health services are currently in transition, which includes the development of a new Australian Mental Health Care Classification [[AMHCC; 1](#_ENREF_1)], the introduction of Activity Based Funding [[ABF; 2](#_ENREF_2)], and formulation of national frameworks for recovery-oriented mental health services [[3](#_ENREF_3), [4](#_ENREF_4)].

Within these frameworks, **recovery-oriented service delivery**: “*… is centered on and adapts to people’s aspirations and needs, rather than people having to adapt to the requirements and priorities of services*” and it has a “…*responsibility to provide evidence-informed treatment, therapy, rehabilitation and psychosocial support that assist in achieving the best outcomes for people’s mental health, physical health and wellbeing*” [[4, p. 26](#_ENREF_4)].

At present, distinctions between sub-acute and non-acute care are independent of the overall care type (e.g., rehabilitation, palliative, psychogeriatric). As detailed by the Australian Institute of Health and Welfare [[5](#_ENREF_5)]:

**Sub-acute care** is: *“… specialised multidisciplinary care in which the primary need for care is optimisation of the* ***patient’s functioning and quality of life*** …”, relating to “…*their whole body or a body part, … the whole person in a social context, … activity limitation and/or participation restriction*” (p. 9) – “… *delivered under the management of or informed by* ***a clinician with specialised expertise*** *in [care type]*” and “… *evidenced by an* ***individualised multidisciplinary management plan****, which is documented in the patient’s medical record*” (p. 10).

By comparison –

**Non-acute (or ‘*maintenance*’) care** is: “… *care in which the primary clinical purpose or treatment goal is* ***support for a patient with impairment, activity limitation or participation restriction due to a health condition****. Following assessment or treatment the patient does not require further complex assessment or stabilisation* ...”, often requiring “… *care over an indefinite period*” (p. 13).

‘Mental Health Care’ is also about to be introduced as a formal, overarching ‘care type’ within Australian health services [[1](#_ENREF_1)], with the following (interim) definition:

**Mental health care** is: *“care in which the primary clinical purpose or treatment goal is* ***improvement in the symptoms and/or psychosocial, environmental and physical function relating to a patient’s mental disorder”****.*

*Mental health care* *is delivered under the management of, or regularly informed by,* ***a clinician with specialised expertise in mental health****;* *is evidenced by* ***an individualised formal mental health assessment*** *and the implementation of* ***a documented mental health plan****; and* *may include* ***significant psychosocial components*** *including family and carer support”* (p. 9).

***ISMHU Establishment***

New South Wales (NSW) Health has had a long-standing commitment to build a comprehensive system of community and inpatient care that would reduce social dislocation and disadvantage for people with a serious mental illness (SMI) and high levels of unmet need [[6](#_ENREF_6)]. Improved access to recovery-focused rehabilitation services that are highly integrated and rigorously evaluated was the primary goal, and in 2005 planning commenced for the establishment of a number of 20-bed sub-acute units across NSW; to-date, eight units have been established (totalling 140 beds), although they vary in their models of care and their typical lengths of stay [[e.g., 7](#_ENREF_7)]. A smaller unit, with similar features to ISMHU, has also been established in Canberra [[8](#_ENREF_8)].

Elsewhere in Australia, other supported residential mental health models and services have been established, such as the Prevention and Recovery Care (PARC) services in Victoria [[e.g., 9](#_ENREF_9)], which are essentially lower intensity, non-acute care services developed in partnership with Community Managed or Non-government Organisations (CMOs/NGOs); there have also been calls for a broader array of non-acute mental health rehabilitation services in NSW [[10](#_ENREF_10)].

In short, and with respect to the broader service context described above, ISMHU is: a publicly funded, specialised, multidisciplinary mental health service providing predominantly sub-acute care, with a specific focus on recovery-oriented clinical rehabilitation, and the delivery of programs, interventions and linkages within an ‘intermediate’ timeframe.

For Hunter New England Mental Health, the capital works program initiated in 2005 provided an opportunity to develop an innovative model of sub-acute care at a level of service delivery that had hitherto not been explored. The overall ISHMU program was designed to operate within the broader framework of an Integrated Recovery-oriented Model (IRM) for mental health services [[11](#_ENREF_11)]; the primary purpose of this program was to address the recovery needs of people with SMI by improving their access to a suite of evidence-based interventions provided within an integrated, multi-layered service context. The ISMHU service model and 6-week program was developed under the stewardship of a committee that included clients, carers, representatives of NGOs and clinical staff from a range of service settings [Frost B, Cant G. *Intermediate Stay Mental Health Unit (ISMHU) - Service Model*, 2010. HNEMH: unpublished, available upon request].

The primary program goals were to improve psychological and physical wellness, enhance personal and interpersonal coping skills, improve daily functioning, enhance social, family and community supports and, thereby, to encourage a new or higher sense of self-management and social inclusion through the attainment of socially valued roles. As detailed in the main text, the Mental Health Recovery Star [[MHRS; 12](#_ENREF_12)] plays a central role in the ISMHU program, both as a collaborative planning, review and assessment tool, and as a general framework for describing programs and encouraging attendance, including a program guide identifying which of the ten MHRS domains are addressed by each of the core programs and electives.

To optimise access, the criteria for ISMHU admission and transfer were kept to a minimum. The primary focus was the recovery needs and priorities of adults aged 16-65 years with a SMI who were *not* acute and considered to be at low risk. As a stand-alone unit, risk had to be manageable. Substance misuse was not an exclusion criterion, although the use of synthetic and other drugs whilst outside of the unit was responsible for some clients being transferred to the acute inpatient unit (9 kilometres away). This option was only exercised as a last resort. A seclusion room was deliberately not included in ISMHU’s design specifications.

***Staffing***

To underpin the ISMHU recovery-oriented model, a staffing profile and roster arrangements were developed that departed from traditional approaches, including: an increased number of occupational therapists, occupational therapy assistants and social workers; a consultant psychiatrist; registrars; a career medical officer; nurses; a psychologist; and a part-time dietician and pharmacist. The increase in allied health professionals was designed to support access to after-hours and weekend interventions (both individual and group) for clients and their families. An NGO peer-link worker (consumer advocate) was also included in ISMHU’s staffing profile. This position operated as part of the team and liaised closely with community-based day programs, supported accommodation services, transitional living, supported employment, supported educational, and General Practitioner (GP) shared care programs. Allied health professionals were rostered to cover weekday shifts from 8am to 8.30pm, with occupational therapists and assistants also covering daytime shifts on weekends. Medical staff were allocated to day shifts during the week, with after-hours and weekend medical coverage provided by the mental health emergency department at the Mater Mental Health unit, Waratah. ISMHU medical staff also generously contributed to after-hours programs and services when required.

The recruitment of skilled rehabilitation clinicians who shared a similar set of values was critical to the program’s success. Promoting recovery through clinical rehabilitation requires a particular values and skill set, supported by a positive team culture, clinical review and supervision processes. Working with clients to achieve higher levels of independence requires a graduated development and exposure to opportunities that often entail risks. The characteristics required by staff included openness, a collaborative focus on the client’s inner resources, understanding of and preparedness to go the extra distance [[13](#_ENREF_13)]. The skill set included empathy, caring, accepting, encouraging, supporting responsible risk taking, and a positive outlook [[13](#_ENREF_13)]. Staff selection procedures focused on a behaviourally-based knowledge of recovery-oriented principles and practices, as well as relationship skills and personal values. A comprehensive training program was developed to support the recruitment strategy.

Fundamental to any care coordination model is consistency in support and approach. This presented a number of challenges for a recovery-oriented model operating across multiple levels of care. To ensure that there was a strong sense of continuity across the 6-week admission, a unit-based coordinator was initially allocated who would work closely with the community-based worker. Thus far, engagement with community-based workers has been variable but this may improve as the role and contribution of the unit is better understood.

***Training Strategy***

The promotion of recovery-oriented practices, and staff training needs and programs, are being increasingly raised as issues for mental health services [[14](#_ENREF_14)]. An extensive information and education strategy was developed to support the introduction of the new ISMHU model of care, which also included mental health services’ staff and key stakeholders in other areas of care delivery. As the effectiveness of such strategies can be dependent on the degree of previous needs-related exposure and rehabilitation experience, a senior rehabilitation clinician was functionally ‘embedded’ in acute inpatient and community-based mental health services, as well as specialist services. In regional areas, senior rehabilitation practitioners performed a similar role. For the CMO/NGO sector, consultants were available on request.

Senior rehabilitation staff were responsible for intervening early and engaging with clients, carers and the clinical team, to develop a recovery-oriented plan that could be relayed to key ISMHU staff well in advance of admission or transfer. This process was critical in ensuring that the focus and duration of the admission was not adversely affected by the post-transfer identification of needs, including accommodation difficulties. In complex medical cases, the ISMHU consultant psychiatrist and medical staff supported the rehabilitation clinician in promptly resolving any treatment issues that may have affected the recovery plan and delayed transfer.

***References***

1. Commonwealth of Australia: *Development of the Australian Mental Health Care Classification - public consultation paper 1.* Canberra: Independent Hospital Pricing Authority, CoA; 2015.

2. Commonwealth of Australia: *Consultation paper on the Pricing Framework for Australian Public Hospital Services 2016-17.* Canberra: Independent Hospital Pricing Authority, CoA; 2015.

3. Commonwealth of Australia: *A national framework for recovery-oriented mental health services: Guide for practitioners and providers.* Canberra: Department of Health and Ageing, CoA; 2013.

4. Commonwealth of Australia: *A national framework for recovery-oriented mental health services: Policy and theory.* Canberra: Department of Health and Ageing, CoA; 2013.

5. Australian Institute of Health and Welfare: *Development of nationally consistent subacute and non-acute admitted patient care data definitions and guidelines. Cat. no. HSE 135.* Canberra: AIHW; 2013.

6. NSW Department of Health: *A New Direction for Mental Health.* Sydney: NSW Health; 2006.

7. Panesar N, Valachova I, Lynch W, Pai N: **Establishing a recovery-focused rehabilitation unit: A case example.** *Aust J Rehabil Couns* 2011, **17:**46-53.

8. Thomas K, Rickwood DJ, Bussenschutt G: **Adult step-up step-down: A sub-acute short-term residential mental health service.** *Int J Psychosoc Rehabil* 2015, **19:**13-21.

9. Lee SJ, Collister L, Stafrace S, Crowther E, Kroschel J, Kulkarni J: **Promoting recovery via an integrated model of care to deliver a bed-based, mental health prevention and recovery centre.** *Australas Psychiatry* 2014, **22:**481-488.

10. Mental Health Coordinating Council: *Submission to NSW Health: Proposal to Pilot a Community Managed Step-up and Home based Outreach (Sub-acute) Mental Health Service in New South Wales (2011-2014).* Sydney: Mental Health Coordinating Council; 2010.

11. Frost BG, Tirupati S, Johnston S, Turrell M, Lewin TJ, Sly KA, Conrad AM: **An Integrated Recovery-oriented Model (IRM) for mental health services: evolution and challenges.** *BMC Psychiatry (In Press)* 2016.

12. MacKeith J, Burns S (Ed.). **Mental Health Recovery Star; User Guide**, Second edition. London: Triangle Consulting and Mental Health Providers Forum; 2010.

13. Borg M, Kristiansen K: **Recovery-oriented professionals: Helping relationships in mental health services.** *J Ment Health* 2004, **13:**493-505.

14. Gilburt H, Slade M, Bird V, Oduola S, Craig TK: **Promoting recovery-oriented practice in mental health services: a quasi-experimental mixed-methods study.** *BMC Psychiatry* 2013, **13:**167.
