# Supplementary material for: Implementation of a recovery-oriented model in a sub-acute Intermediate Stay Mental Health Unit (ISMHU)
Source: BMC Health Serv Res. 2017 Jan 3;17:2. doi: 10.1186/s12913-016-1939-8 (PMC5210223; doi:10.1186/s12913-016-1939-8)
Supplement: Additional file 2: — Mental Health Recovery Star (MHRS) Dimensionality – Explorations using data from the Intermediate Stay Mental Health Unit. (DOCX 84 kb) [file 12913_2016_1939_MOESM2_ESM.docx]

**Supplementary Document 2 (December, 2016): *“Mental Health Recovery Star (MHRS) Dimensionality – Explorations using data from the Intermediate Stay Mental Health Unit (ISMHU, Hunter New England Mental Health Services, Newcastle, Australia)”***

***Background***

The Mental Health Recovery Star [[MHRS; 1](#_ENREF_1)] has received a somewhat mixed reception, depending on the evaluator’s views about its primary purpose. For those who view it as a multi-dimensional, collaborative and client-centred tool for assessing current recovery experiences and plans, it has generally been well received [e.g., [1](#_ENREF_1), [2](#_ENREF_2), [3-5](#_ENREF_3)]. On the other hand, for those seeking a new/alternative ‘objective’ or ‘validated’ recovery-focused ‘outcome measure’, it has been less well received [e.g., [6](#_ENREF_6), [7-9](#_ENREF_7)].

Some criticisms of the MHRS simply reflect its stage of development, and relate primarily to the absence of sufficient research or clinical evidence to-date [[6](#_ENREF_6), [8](#_ENREF_8)]. Implied criticisms about the lack of consumer input [[8](#_ENREF_8)] during the MHRS’s development are essentially mistaken [[see 4](#_ENREF_4)]; likewise, concerns about the time required for MHRS administration [[8](#_ENREF_8)] fail to take into account that it is a collaborative tool designed to be embedded within routine clinical practice.

It is acknowledged that we genuinely need additional information about the utility and psychometric properties of the MHRS [[10](#_ENREF_10)]. However, while these aspects of the MHRS have been questioned by some researchers [e.g., [6](#_ENREF_6), [7-9](#_ENREF_7)], their evaluations and criticisms have generally been poorly or narrowly framed [[2](#_ENREF_2), [3](#_ENREF_3), [10](#_ENREF_10)].

Recently, there have also been a range of constructive discussions and suggestions about the day-to-day application of the MHRS and its measurement properties [e.g., [3](#_ENREF_3), [10](#_ENREF_10)]. Notwithstanding, there have been few analyses of the factor structure of the MHRS [[e.g., 2](#_ENREF_2)] and an optimal (or preferred) scoring scheme has yet to be identified. This document briefly examines the dimensionality and psychometric properties of the MHRS using available data from a recent service evaluation project.

***Data Sources and Analysis***

One-hundred and fifty-four (*N* = 154) clients were admitted to the Intermediate Stay Mental Health Unit (ISMHU, Newcastle, Australia) during the first 16 months of its operation (mean age: 37.2 [SD 10.2] years; males: 72.1%). An index admission was identified for each of these clients, with 10 clients having more than one admission during the period. MHRS ratings were available for 131 clients on admission and 97 clients at discharge, with 94 sets of paired ratings. As detailed below, some analyses were conducted using all available sets of ratings, while others were restricted to clients with both admission and discharge ratings.

**Principal Component Analysis of Raw MHRS Item Ratings.** Table S1 presents the findings from an initial series of T-technique (Total variation) principal component analyses using raw MHRS ratings. Three data sets were examined in these analyses: Set A, all available MHRS ratings; Set B, excluding ratings of “10” for the Addictive behaviour and Responsibilities domains, which the MHRS automatically assigns when that area is not considered to be a problem; and Set C, ratings from clients with complete sets of MHRS admission and discharge ratings. Multiple, overlapping data sets were examined in these analyses to assess the consistency of the observed factor structure and loadings, particularly in comparison to Set C, which provided the basis for subsequent change and related analyses. While a small second principal component emerged in the Set A and Set C analyses (with respective eigenvalues of 1.035 and 1.072, which was associated primarily with the Addictive behaviour item), there was clearly a very strong and consistent first principal component in all analyses (with eigenvalues ranging from 4.916 to 5.214, and all items loading above 0.40); consequently, based on this analysis of the raw MHRS item ratings, a single overall MHRS score utilising all 10 item domains would be appropriate.

| **Table S1.** *Factor loadings on first principal component from selected T-technique (Total variation) analyses of Mental Health Recovery Star (MHRS) ratings* | | | | |
| --- | --- | --- | --- | --- |
| **MHRS item** | | **Data set**: | | |
|  |  | **A: All available ratings**  (Admission: *N* = 131;  Discharge: *N* = 97) | **B: Excluding selected ratings**  (Admission: *N* = 131;  Discharge: *N* = 97) | **C: Ratings from clients with both assessments**  (Admission: *N* = 94;  Discharge: *N* = 94) |
|  |  |  |  |  |
| 9 | Identity and self-esteem | .832 | .841 | .832 |
| 10 | Trust and hope | .783 | .783 | .813 |
| 4 | Social networks | .781 | .778 | .802 |
| 1 | Managing mental health | .751 | .750 | .763 |
| 2 | Physical health and self-care | .740 | .741 | .721 |
| 3 | Living skills | .713 | .714 | .720 |
| 5 | Work | .654 | .635 | .637 |
| 6 | Relationships | .653 | .655 | .624 |
| 8 | Responsibilities | .605 | .717 | .653 |
| 7 | Addictive behaviour | .402 | .565 | .405 |
|  |  |  |  |  |
|  | Cumulative % of variance | 49.16 | 52.14 | 50.03 |
| *Note*: Two principal components had eigenvalues greater than 1.0 for the Set A (4.916, 1.035) and Set C (5.003, 1.072) analyses. For the Set B analysis, clients with admission ratings of “10” on Addictive behaviour (*N* = 31) or Responsibilities (*N* = 28) had those items excluded (i.e., the MHRS value automatically assigned when that area is not a problem), together with their corresponding discharge ratings; in the Set B analysis, only the first principal component had an eigenvalue greater than 1.0 (5.214). | | | | |

**Principal Component Analysis of Individually Standardized MHRS Item Ratings.** Since the MHRS is a self-report collaborative tool rated primarily by individual clients (with clinician guidance), it is likely that, at least initially, clients would have limited knowledge about the measure or how others would interpret or use the particular (1 to 10) rating scales (although detailed MHRS ladders of change and associated anchors are provided). Consequently, and given its overall focus on self-perceived capacity to manage independently, the MHRS potentially has more relevance for the assessment of how individuals change (i.e., as a serial and relative, or within-subject measure) than for direct comparisons between clients.

To remove some of the variance in ratings due to differences between individuals in overall scale usage, we *individually standardised* MHRS ratings for data Set C (*N* = 188 sets of ratings); the grand mean and SD across the 20 (admission and discharge) ratings per person were used as the reference point for standardisation (i.e., z-score conversion) – that is, effectively centring each person’s ratings around their own (multi-timepoint) average rating. Table S2 presents the findings from a T-technique principal component analysis of these *individually standardised* ratings, using an oblique rotation. In this instance, three factors accounted for 49.7% of the item variance (initial eigenvalues: 2.533, 1.311, and 1.129). The factor loadings generally suggested that MHRS items could be uniquely allocated to one of these three factors, with only one item (Responsibilities) potentially having mixed loadings (on the second and third factors). Factor 1 – labelled ‘**Symptom management & functioning**’ – comprised four items (Physical health and self-care; Managing mental health; Work; and Living skills); Factor 2 – labelled ‘**Self-belief**’ – also comprised four items (Addictive behaviour; Identity and self-esteem; Trust and hope; and Responsibilities); while Factor 3 – labelled ‘**Social connection**’ – comprised two items (Relationships; and Social networks).

Apart from their general face validity, the factors identified in Table S2 also appear to map reasonably well onto several of the broad (‘C.H.I.M.E.’) recovery processes identified by Leamy and colleagues [[11](#_ENREF_11), [12](#_ENREF_12)] and by earlier researchers [[e.g., 13](#_ENREF_13)]. For example: Factor 3 maps onto ‘**C**onnectedness’ (Category 1); Factor 2 maps jointly onto ‘**H**ope and Optimism’ and ‘**I**dentity’ (Categories 2 and 3); and Factor 1 maps onto ‘**M**eaning in Life’ (Category 4); with ‘**E**mpowerment’ (Category 5, e.g., personal responsibilities and control) having limited representation as a separate factor in the MHRS. Likewise, the MHRS factors are not inconsistent with the taxonomy of “five broad superordinate recovery dimensions” suggested by Whitley and Drake [[14](#_ENREF_14)]: Factor 1 maps onto their Clinical, Physical and Functional dimensions, Factor 2 onto their Existential dimension, and Factor 3 onto their Social dimension of recovery.

| **Table S2.** *Factor loadings from a T-technique (Total variation) principal component analysis of individually standardized Mental Health Recovery Star (MHRS) item ratings, using an oblique rotation* | | | | |
| --- | --- | --- | --- | --- |
| **MHRS item** | | **Data set C**: Clients with both assessments  (Admission: *N* = 94; Discharge: *N* = 94) | | |
|  |  | Factor 1:  **Symptom management & functioning** | Factor 2:  **Self-belief** | Factor 3:  **Social**  **connection** |
|  |  |  |  |  |
| 2 | Physical health and self-care | .657 | -.028 | -.172 |
| 1 | Managing mental health | .626 | .241 | -.093 |
| 5 | Work | .621 | .017 | .297 |
| 3 | Living skills | .608 | -.072 | -.079 |
| 7 | Addictive behaviour | -.209 | .760 | .238 |
| 9 | Identity and self-esteem | .235 | .629 | -.071 |
| 10 | Trust and hope | .198 | .613 | -.085 |
| 8 | Responsibilities | -.120 | .397 | -.354 |
| 6 | Relationships | -.042 | -.099 | -.840 |
| 4 | Social networks | .280 | .094 | -.635 |
| *Note*: Item ratings were standardised using each individual’s grand mean and grand SD (i.e., across 20 MHRS item ratings – from admission and discharge). The three-factor solution accounted for 49.7% of the item variance (initial eigenvalues: 2.533, 1.311, and 1.129). | | | | |

**Calculation of Subscale and Overall MHRS Scores.** For each phase (i.e., admission or discharge), MHRS subscale scores were derived by averaging (unadjusted) item ratings for the four, four, and two items, respectively, allocated to each factor (see Table S2). Likewise, overall MHRS scores were obtained by averaging responses to the 10 items. Thus, all calculated MHRS scores were expressed using the same, common (1 to 10) metric as the individual items. Overall correlations (*p* < .001) between the resulting MHRS subscales (Set A, *N* = 228 sets of ratings) were: Factors 1 & 2, *r* = 0.652; Factors 1 & 3, *r* = 0.659; and Factors 2 & 3, *r* = 0.602.

**Internal Consistency Analysis.** Table S3 reports internal consistency coefficients (Cronbach’s α) for the MHRS subscales and the overall MHRS score for Set A (*N* = 228 sets of ratings) and Set C (*N* = 188 sets of ratings). For Set A, the internal consistency coefficients were: Factor 1 (Symptom management & functioning), α = 0.784; Factor 2 (Self-belief), α = 0.744; Factor 3 (Social connection), α = 0.687; and overall MHRS score, α = 0.877; with all internal consistency coefficients regarded as acceptable. The Cronbach’s α for the overall MHRS score is comparable to the α = 0.85 value reported by Dickens et al. [[2](#_ENREF_2)], although their exploratory factor analysis produced two reasonably discordant factors (representing internal versus external management/relationships).

Table S3 also presents correlations between admission and discharge ratings for individual items, subscales and overall MHRS scores (for Set C). Across the 6-week admission, item level correlations between admission and discharge ratings ranged from *r* = 0.461 (for Physical health and self-care) to *r* = 0.624 (for Responsibilities), with a correlation of *r* = 0.575 for overall MHRS scores.

| **Table S3.** *Internal consistency coefficients (Cronbach’s α) for selected Mental Health Recovery Star (MHRS) subscales and overall, and correlations between admission and discharge ratings* | | | | |
| --- | --- | --- | --- | --- |
| **MHRS**  **Subscale / item** | | **Data set**: | | |
|  |  | **A: All available ratings**  (Admission: *N* = 131;  Discharge: *N* = 97) | **C: Ratings from clients with both assessments**  (Admission: *N* = 94; Discharge: *N* = 94) | |
|  |  | Internal  Consistency | Internal  Consistency | Correlation between Admission and Discharge ratings |
| **Symptom management & functioning** | | .784 | .786 | **.632** |
| 2 | Physical health and self-care |  |  | .461 |
| 1 | Managing mental health |  |  | .529 |
| 5 | Work |  |  | .608 |
| 3 | Living skills |  |  | .567 |
|  | |  |  |  |
| **Self-belief** | | .744 | .777 | **.629** |
| 7 | Addictive behaviour |  |  | .595 |
| 9 | Identity and self-esteem |  |  | .592 |
| 10 | Trust and hope |  |  | .616 |
| 8 | Responsibilities |  |  | .624 |
|  | |  |  |  |
| **Social connection** | | .687 | .714 | **.539** |
| 6 | Relationships |  |  | .580 |
| 4 | Social networks |  |  | .480 |
|  |  |  |  |  |
| **Overall** **score** (10 items) | | .877 | .880 | **.575** |
| *Note*: The mean interval between admission and discharge ratings was 37.9 days (SD = 17.4). | | | | |

***Recommendations***

In short, a single factor solution does a reasonably good job at accounting for variation among the (unadjusted) item ratings (see Table S1), providing strong justification for forming an overall MHRS score based on (averaging) the 10 items. However, to facilitate concurrent evaluation of some of the potentially useful underlying recovery components, it also seems reasonable to *additionally* construct some correlated subscale scores (see Tables S2 and S3); the latter may be particularly useful for examining change (and differential predictive utility), given the manner in which they were initially derived (i.e., using individually standardised MHRS item ratings, which did not distort differences across phases).

***Acknowledgements***

Some of the material reported here was initially presented at the Annual Society for Mental Health Research (SMHR) conference, Adelaide, South Australia (December 3rd, 2014): Lewin, T. J., Sly, K. A., Conrad, A. M., & Frost, B. (2014). *Recovery patterns during a 6-week admission to a sub-acute Intermediate Stay Mental Health Unit (ISMHU)*.

***References***

1. MacKeith J, Burns S (Ed.). **Mental Health Recovery Star; User Guide**, Second edition. London: Triangle Consulting and Mental Health Providers Forum; 2010.

2. Dickens G, Weleminsky J, Onifade Y, Sugarman P: **Recovery Star: Validating user recovery.** *The Psychiatrist* 2012, **36:**45-50.

3. Tickle A, Cheung N, Walker C: **Professionals' perceptions of the Mental Health Recovery Star.** *Mental Health Review Journal* 2013, **18:**194-203.

4. Onifade Y: **The mental health recovery star.** *Mental Health and Social Inclusion* 2011, **15:**78-87.

5. Lloyd C, Williams PL, Machingura T, Tse S: **A focus on recovery: using the Mental Health Recovery Star as an outcome measure.** *Advances in Mental Health* 2016, **14:**57-64.

6. Burgess P, Pirkis J, Coombs T, Rosen A: **Assessing the value of existing recovery measures for routine use in Australian mental health services.** *Aust N Z J Psychiatry* 2011, **45:**267-280.

7. Killaspy H, White S, Taylor TL, King M: **Psychometric properties of the Mental Health Recovery Star.** *Br J Psychiatry* 2012, **201:**65-70.

8. Sklar M, Groessl EJ, O'Connell M, Davidson L, Aarons GA: **Instruments for measuring mental health recovery: A systematic review.** *Clin Psychol Rev* 2013, **33:**1082-1095.

9. Beazley PI: **(Letter) The Recovery Star: is it a valid tool?** *The Psychiatrist* 2011, **35:**196-197.

10. Recovery Star Research Seminar: *Report of Recovery Star Research Seminar - June 14, 2013 (Chaired by Prof. Nick Manning).* Institute of Mental Health (Nottingham) and Triangle Consulting; 2013.

11. Le Boutillier C, Leamy M, Bird VJ, Davidson L, Williams J, Slade M: **What does recovery mean in practice? A qualitative analysis of international recovery-oriented practice guidance.** *Psychiatr Serv* 2011, **62:**1470-1476.

12. Bird VJ, Le Boutillier C, Leamy M, Larsen J, Oades LG, Williams J, Slade M: **Assessing the strengths of mental health consumers: A systematic review.** *Psychol Assess* 2012, **24:**1024-1033.

13. Andresen R, Oades L, Caputi P: **The experience of recovery from schizophrenia: towards an empirically validated stage model.** *Aust N Z J Psychiatry* 2003, **37:**586-594.

14. Whitley R, Drake RE: **Recovery: a dimensional approach.** *Psychiatr Serv* 2010, **61:**1248-1250.
